# Telemedicine for Preventing and Treating Pressure Injury After Spinal Cord Injury: Systematic Review and Meta-analysis

**DOI:** 10.2196/37618

**Published:** 2022-09-07

**Authors:** Guilian Chen, Tong Wang, Lirong Zhong, Xinghui He, Chunxia Huang, Yingmin Wang, Kun Li

**Affiliations:** 1 Sun Yat-sen Memorial Hospital of Sun Yat-sen University Guangzhou China; 2 School of Nursing Sun Yat-Sen University Guangzhou China

**Keywords:** spinal cord injury, pressure injury, telemedicine, systematic reviews, meta-analyses, network meta-analyses, review, spinal injury, spinal cord, pressure injury, injury

## Abstract

**Background:**

Pressure injury is a common complication after a spinal cord injury. Long-term multidisciplinary follow-up is difficult after such patients have been discharged. Telemedicine promises to provide convenient and effective support for the prevention and treatment of pressure injury, but previous attempts to demonstrate that have produced inconsistent results.

**Objective:**

The aim of this study is to evaluate the effectiveness of telemedicine in preventing and treating pressure injury among community-dwelling patients with spinal cord injury, and determine which telemedicine form is more effective.

**Methods:**

This systematic review was performed according to the PRISMA-NMA (Preferred Reporting Items for Systematic Reviews and Meta-Analyses extension for Network Meta-Analysis) standards. Ten databases were searched to identify randomized controlled trials and quasi-experimental studies related to the effectiveness of telemedicine intervention in patients with spinal cord injury. Two researchers worked independently and blindly selected studies, extracted data, and assessed the risk of bias. The results were described as relative risk (RR) and weighted mean difference and 95% CI.

**Results:**

The 35 studies comprised 25 randomized controlled trials and 10 quasi-experimental studies involving 3131 patients. The results showed that telemedicine can significantly (*P*<.05) reduce the incidence of pressure injury (RR 0.24, 95% CI 0.14-0.41; *P*<.05; *I*^2^=0%), promote faster healing (RR 0.73, 95% CI 0.62-0.85; *P*<.05; *I*^2^=0%), and yield lower scores on the pressure ulcer scale of healing (weighted mean difference=–1.98, 95% CI –3.51 to –0.46; *P*<.05; *I*^2^=0%). Cumulative ranking estimates showed that combining telemedicine with conventional intervention (93.5%) was the most effective approach.

**Conclusions:**

Telemedicine is a feasible way to prevent pressure injury among patients with spinal cord injuries. It can decrease the incidence and severity of pressure injury and accelerate patients’ healing without imposing economic burden. It is best used in tandem with other, more conventional interventions. Due to the limited quality and quantity of included studies, large-scale and well-designed randomized controlled trials are warranted.

## Introduction

Spinal cord injury (SCI) is a disabling and costly disease, the incidence of which is increasing year by year. The incidence of SCI is estimated to be between 12 and 65 cases per million globally [1] and between 13 and 60 cases per million in China [2]. More than 20% of patients with SCI develop pressure injury as a result of motor and sensory dysfunction, limited body movement, or the long period of time spent in a bed or wheelchair [3]. The daily cost of pressure injury treatment per adult patient ranges from €1.71 (US $1.70) to as much as €470 (US $468.31), and the cost of treating severe pressure injury is even higher [4]. In addition, 7%-8% of deaths among patients with SCI are directly attributable to pressure injury [5]. Pressure injury seriously affects the quality of life of patients with SCI and places a heavy care burden and economic burden on their families and society [6].

There are well-understood measures that can reduce the incidence of pressure injury, and prevention is more cost-effective than treatment [7]. However, most countries have insufficient medical resources, and particularly insufficient professional expertise in the community [8], to provide the necessary long-term and multidisciplinary follow-up of community-dwelling patients with SCI. That results in many obstacles to preventing and treating pressure injury. Today, however, it is becoming more feasible to provide medical services including diagnosis and information about self-care remotely through a variety of communication technologies, including video consultation via mobile apps [9,10]. This has been applied to the prevention and treatment of pressure injury among community-dwelling patients with SCI, but its effectiveness and safety remains inadequately confirmed because systematic studies vary in their sample sizes and conclusions.

Until now, there has been no systematic review of the applicability of telemedicine in preventing and treating pressure injury among community-dwelling patients with SCI. That motivated this systematic review and network meta-analysis. Network meta-analysis can assess both direct and indirect evidence [11]. The biggest advantage is allowing for the simultaneous inclusion of multiple pairwise comparisons in a series of different interventions and ranking the interventions. Therefore, systematic review and network meta-analysis was used to evaluated the effectiveness of telemedicine on the prevention and treatment of pressure injury among community-dwelling patients with SCI, and determine which telemedicine form is more effective, to provided evidence useful for clinical practice.

## Methods

### Study Design and Search Strategy

This systematic review and network meta-analysis was performed according to the PRISMA-NMA (Preferred Reporting Items for Systematic Reviews and Meta-Analyses extension for Network Meta-Analysis) standards [12]. It was registered in the PROSPERO database (International Prospective Register of Systematic Reviews; ID: CRD42020194061).

The databases searched were the China National Key Information corpus, Wanfang, CBM, VIP, Embase, PubMed, Cochrane Library, Web of Science, Scopus, and ProQuest. The dates searched were from establishment of each database to September 30, 2021. Multimedia Appendix 1 shows the keywords used to search each corpus. Keywords and search strategy were designed by the first author, then reviewed by a librarian. Other clinical trial registration websites (Science-paper Online, Open Grey, ClinicalTrials.gov, and China’s Clinical Trial Registry) were searched manually, and references to related papers and reviews were followed up.

Only randomized controlled trials (RCTs) and quasi-experimental studies were included in the systematic review, and only RCTs were included in the network meta-analysis. Beyond that, 4 other criteria were applied.

1. Participants: community-dwelling persons with an SCI.
2. Interventions: complete or partial telemedicine intervention. In complete telemedicine intervention, there was no face-to-face contact during the trial, only telemedicine intervention by telephone, video, or mobile app. Treatment involving only one form of telemedicine intervention was designated as a single complete telemedicine intervention, while therapy combining two or more forms of telemedicine intervention was called a mixed complete telemedicine intervention. Partial telemedicine intervention designated treatment combining telemedicine with a nontelemedicine intervention (such as an outpatient follow-up visit or a home visit).
3. Controls: The “no telemedicine” cases included nontelemedicine intervention and also health guidance only before discharge treated as a blank control. A second type of control was where there was another group treated differently from the experimental group, such as when the experimental group used video and the control group used the telephone. A third case was self-control studies with no control group.
4. Outcomes: Primary and secondary outcomes were considered. The primary outcomes were the incidence of pressure injury, the rate of healing of the pressure injury, and pressure injury severity (size, depth, and Pressure Ulcer Scale for Healing [PUSH]). Any economic data reported were treated as a secondary outcome.

Certain reports had to be excluded, for example, academic meeting abstracts or papers without full text; papers published repeatedly; and papers for which adequate data could not be obtained even after contacting the authors.

### Data Extraction

Two authors (the first and the second author) worked independently and blindly to screen titles, abstracts, and full texts, and select studies applying the inclusion and exclusion criteria. Any disagreements were resolved by discussion or by consulting the corresponding author. EndNote X9 software (Clarivate) was first used to exclude duplicates. Then, reading the title and abstract was enough to exclude clearly irrelevant papers. Finally, reading the full text allowed us to determine whether or not a study should be included. If necessary, authors were contacted by email or telephone for further information.

The first and second authors also worked independently and blindly to extract data and assess the risk of bias, again consulting the corresponding author if necessary. The data extracted included each study’s characteristics, participant characteristics, intervention and control treatments, and outcomes. The Cochrane risk of bias tool [13] was used to assess the RCTs, and the Joanna Briggs Institute critical appraisal tool [14] was used with the quasi-experimental studies.

### Statistical Analysis

*I*^2^ statistic was used to evaluate the consistency of the results of included studies, with 25%, 50%, and 75% representing low, moderate, and high heterogeneity, respectively [15]. The fixed-effect model was used when the heterogeneity was acceptable (*I*^2^ ≤50%, *P*>.10), otherwise the random effect model was used. If the heterogeneity was still too large after subgroup analysis or sensitivity analysis, if the number of studies was too small, or if the data could not be synthesized, only descriptive analysis was performed. Dichotomous data were analyzed using relative risk (RR) and 95% CIs. Continuous data were analyzed using weighted mean difference (WMD) where the same tools were used, and standardized mean difference where different studies used different tools. When *P*<.05, the difference between the two groups was statistically significant.

Song [16] has proposed that network meta-analysis should satisfy hypotheses about homogeneity, similarity, and consistency. Otherwise, the nonconformity needs to be explained, or network meta-analysis should not be performed. The evaluation and treatment of the homogeneity requirement is the same as with the heterogeneity of traditional direct comparison meta-analysis. There is no recognized statistical test for verifying the similarity hypothesis, so it must be evaluated based on the characteristics of the included studies. The inconsistency model, node splitting, and inconsistency factors are commonly used to evaluate consistency, with *P*>.05 indicating good consistency in a closed loop if the 95% CI starting point of the inconsistency factor was 0, indicating that the direct and indirect evidence was consistent. RR, WMD, and standardized mean difference were also computed with their 95% CIs. The surface under the cumulative ranking (SUCRA) curve was used to calculate the ranking probabilities of the treatments. The SUCRA values range from 0% to 100%, and the higher the value, the better the result. Comparison-adjusted funnel plots were also used to assess the potential for small study effects.

## Results

### Study Selection

**Figure 1:**
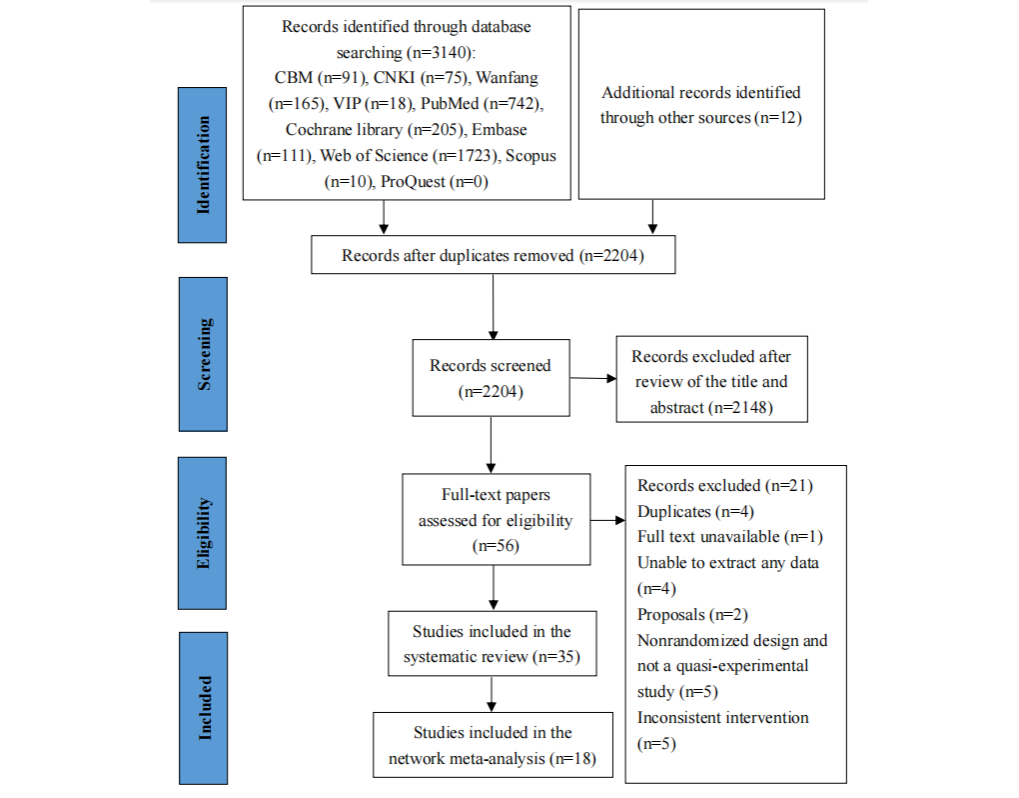
Flowchart for search and selection of the included studies.

The search found 3152 studies. Of those, 948 duplicates were excluded through EndNote. Reading the titles and abstracts of 2204 reports led to 2148 being excluded as irrelevant. Finally, 56 studies were screened in full text, of which 21 were excluded and 35 were finally included (Figure 1).

The 35 studies included 25 RCTs [7,17-40] and 10 quasi-experimental studies [41-50] (Multimedia Appendix 2). The 3131 subjects were community-dwelling patients with SCI, aged 18-96 years old. Of 3131 patients, 2226 (71.1%) were male. Trauma was the most common cause of injury. The top specific causes were traffic accidents, fall from height, fall with heavy objects, and other fall. The study durations ranged from 1 week to 2 years. In most of the studies, nurses served as the main researcher managing diet and nutrition, elimination, and pressure injury, and preventing other complications. They also provided related education and guidance.

Mixed complete telemedicine interventions mainly used the WeChat app and telephone. The average utilization was about 1 hour every day to answer questions, once weekly to convey relevant knowledge, and perhaps a weekly face-to-face video chat if necessary. Telephone calls were made on average once per month, when needed. Single complete telemedicine interventions were mainly delivered via telephone. The average frequency was about once per week. Partial telemedicine intervention was usually a combination of telephone or video telemedicine with outpatient follow-up or home visits. The frequency was about once per week by telephone or face-to-face video, and once per month for outpatient follow-up or home visit. The main form of nontelemedicine intervention was outpatient follow-up or home visit. The frequency was about once per month. The blank control group only received health education before discharge, but the patients could call a medical professional when they needed help.

### Bias Assessment

The overall quality of the studies included was categorized as acceptable. Approximately half of the studies reported randomization, but some reports lacked details about any allocation blinding, which could cause potential selection bias. No study was judged as “low risk” in terms of performance bias because it is very difficult to blind patients in telemedicine intervention trials. About one-quarter of the studies blinded the outcome assessors. There was no evidence of attrition bias, selective reporting bias, and other bias in any of the included studies (Multimedia Appendix 3). In the quasi-experimental studies, item 3 was judged to be “not applicable” to 4 studies [41,46,47,50] because they were self-controlled. Item 7 was judged as “unclear” in 4 studies [44,45,48,49]. The other items all received a “yes” (see Multimedia Appendix 4 for details).

### Meta-analysis and Descriptive Analysis Results

Overall, 27 studies [17-21,23-29,31-35,37-40,42-45,48,49] reported the incidence of pressure injury among their community-dwelling subjects. Among the studies, 18 were RCTs [17-21,24-29,31-35,37,38] analyzed by network meta-analysis, 5 were quasi-experimental studies [42-44,48,49] analyzed by meta-analysis, and 4 [23,39,40,45] could not be combined for descriptive analysis.

The meta-analysis showed that the incidence of pressure injury was significantly lower in the telemedicine intervention group (n=468; RR 0.24, 95% CI 0.14-0.41; *P*<.05; *I*^2^=0%, fixed-effects model; Multimedia Appendix 5). The other 4 studies which could not be combined also found that the incidence of pressure injury in the intervention group was lower than in the control group (*P<*.05).

**Figure 2:**
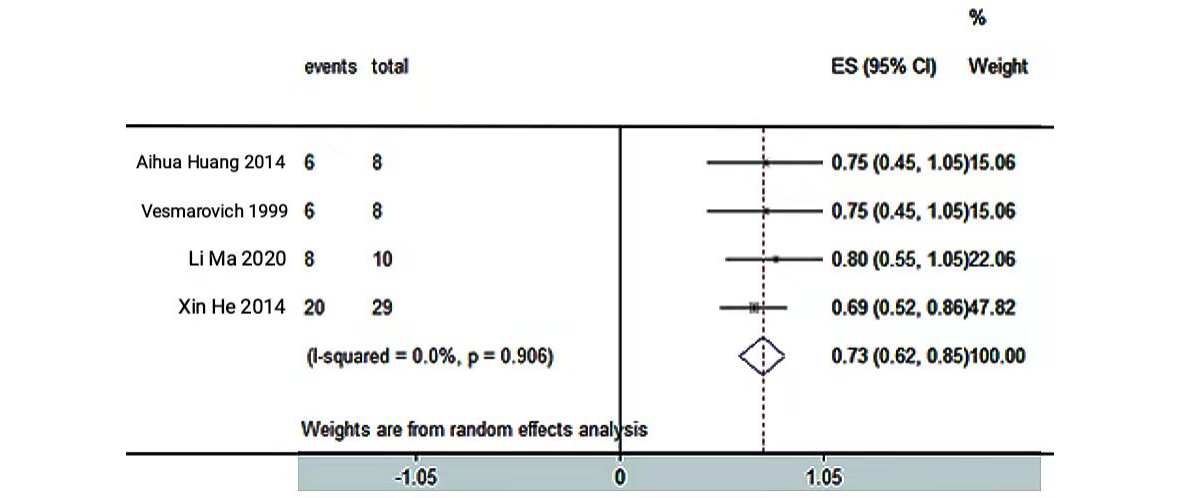
The effectiveness of telemedicine on the healing rate of pressure injury. ES: effect size.

A total of 9 studies [17,21,22,41,44,46,47,49,50] reported the rate of pressure injury healing. There were 3 RCTs [17,21,22] and 6 quasi-experimental studies [41,44,46,47,49,50]. Due to the limited sample size, 4 studies [41,46,47,50] (self-controlled) were classified as the telemedicine intervention group. The other 5 [17,21,22,44,49] were descriptive because the data could not be combined. The meta-analysis showed that the rate of pressure injury healing was significantly faster in the telemedicine intervention group (n=55; RR 0.73, 95% CI 0.62-0.85; *P*<.05; *I*^2^=0%, fixed-effects model; Figure 2). In the other 5 studies without meta-analysis, 14 patients in the telemedicine intervention group healed, along with 9 patients in the control group.

**Figure 3:**
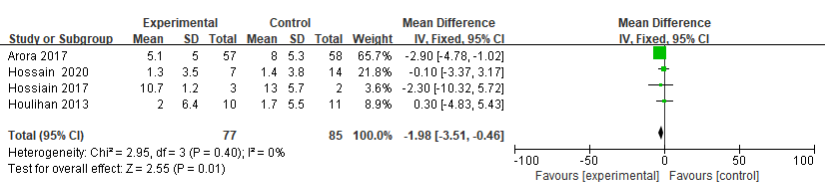
Comparison of the effectiveness of telemedicine and control on PUSH scores. PUSH: Pressure Ulcer Scale for Healing.

A total of 4 RCTs [7,19,27,36] reported on the severity of the pressure injury studied. The meta-analysis showed that patients in a telemedicine intervention group tended to have lower PUSH scores (n=162; WMD=–1.98, 95% CI –3.51 to –0.46; *P*=.01; *I*^2^=0%, fixed-effects model; Figure 3). Although one study [7] found no significant difference in the improvement of pressure injury area and depth, the improvement in the other telemedicine groups was significantly better than in the corresponding control group.

Only one study (an RCT) [7] reported economic data. The incremental cost-effectiveness ratio was 2306 Indian rupees (approximately US $130) per 1 cm^2^ reduction in pressure injury area and 44,915 Indian rupees (US $2523) per additional quality-adjusted life year. This result shows that the telemedicine intervention was cost-effective, at least in India.

### Network Meta-analysis Results

**Figure 4:**
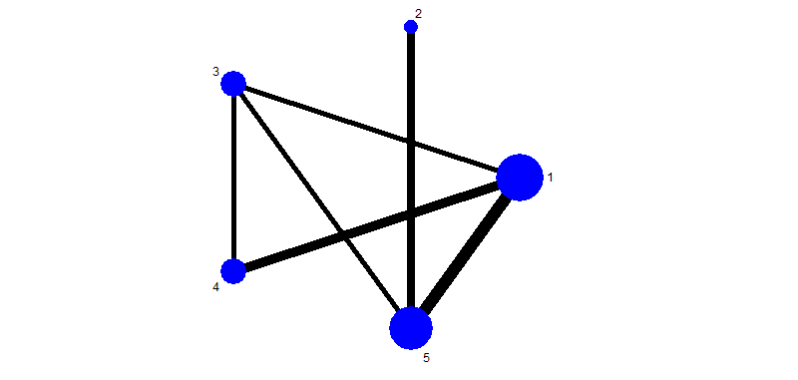
Network meta-analysis of eligible comparisons for incidence. 1: blank control; 2: nontelemedicine intervention; 3: single complete telemedicine intervention; 4: mixed complete telemedicine intervention; 5: partial telemedicine intervention.

A total of 18 RCTs [17-21,24-26,28,29,31-35,37,38,45] involving 5 forms of intervention were included in the network meta-analysis. A network plot for the incidence of pressure injury was produced using the STATA software package (version 14.0; StataCorp LLC). Nodes indicated treatments, with the size of each node proportional to the number of observations in the sample. The thickness of the lines was proportional to the number of studies directly comparing a pair of treatments. Two triangles were formed with the interventions in this study: triangle 134 and triangle 135. The most numerous comparable studies involved partial telemedicine intervention with a blank control (Figure 4).

A consistency test did not identify statistically significant inconsistency (*X*^2^=3.76，*P*=.15). The loops were consistent, since their 95% CI included 0. Node splitting showed no statistically significant difference between the direct and indirect estimate of the summary effect (see Multimedia Appendix 6 for details).

**Figure 5:**
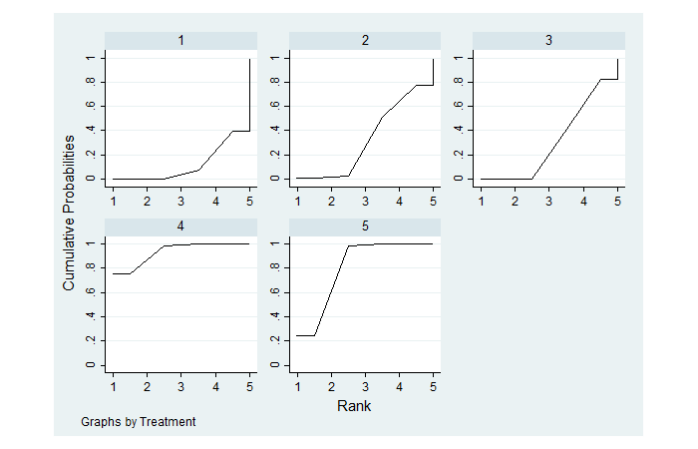
The surface under the cumulative ranking estimate. 1: blank control; 2: nontelemedicine intervention; 3: single complete telemedicine intervention; 4: mixed complete telemedicine intervention; 5: partial telemedicine intervention.

The SUCRA estimates (Figure 5) and the SUCRA value (Multimedia Appendix 7) show that mixed complete telemedicine intervention was the best form of intervention for reducing the incidence of pressure injury. Mixed complete telemedicine intervention (93.5%) was better than partial telemedicine intervention (80.5%), which was better than nontelemedicine intervention (32.7%), single complete telemedicine intervention (31.7%), and blank control (11.7%) (see Table 1 for details). The comparison-adjusted funnel plot was basically symmetrical, indicating that the possibility of publication bias was small (Multimedia Appendix 8).

**Table 1:** The effectiveness of telemedicine in preventing pressure injury according to the network meta-analysis.

MCTI^a^	PTI^b^	SCTI^c^	NTI^d^	Blank control
MCTI	1.34 (0.59-3.06)	3.52 (1.95-6.36)	3.35 (1.07-10.48)	4.63 (2.16-9.93)
0.75 (0.33-1.70)	PTI	2.62 (1.29-5.36)	2.50 (1.13-5.49)	3.45 (2.09-5.71)
0.28 (0.16-0.51)	0.38 (0.19-0.78)	SCTI	0.95 (0.33-2.75)	1.31 (0.67-2.59)
0.30 (0.10-0.93)	0.40 (0.18-0.88)	1.05 (0.36-3.04)	NTI	1.38 (0.54-3.52)
0.22 (0.10-0.46)	0.29 (0.18-0.48)	0.76 (0.39-1.50)	0.72 (0.28-1.84)	Blank control

^a^MCTI: mixed complete telemedicine intervention.

^b^PTI: partial telemedicine intervention.

^c^SCTI: single complete telemedicine intervention.

^d^NTI: nontelemedicine intervention.

## Discussion

### Principal Findings

This systematic review and network meta-analysis results show that telemedicine intervention can reduce the incidence and severity of pressure injury and improve the rate of healing of such injuries without increasing the medical economic burden on community-dwelling patients with SCI. In addition, the results indicate that combining telemedicine with conventional interventions is the most effective form of intervention for preventing pressure injury.

Although the overall quality of the studies was regarded as acceptable, none were able to blind the participants and personnel. Some reports mentioned random sequence generation, allocation concealment, and blinding, but without specifics. That may relate to the space limitations of journal publications or the design of the experiments. This review included only studies published in Chinese or English, of which many were Chinese. This may be related to the Quality Nursing Service demonstration project launched by China’s National Health Commission in early 2010 [51] and to its “Internet+” policy implemented in 2016 [52]. More than 10 studies in this systematic review were conducted using QQ and WeChat, which are Chinese social media platforms that support sending text, pictures, and videos, and support multiperson group chats via the internet. Such social media software, which have a high penetration rate, broad mass base, rich features, and no extra charge, are likely to be central to the future development of telemedicine.

The results show that telemedicine intervention can reduce the incidence and severity of pressure injury. As part of rehabilitation, patients with SCI were usually educated in preventive skin care techniques, but they are often not continued after discharge [53,54]. Contacting former patients in the community through telemedicine can improve compliance [36], but the prevention and treatment of pressure injury after an SCI involves several medical disciplines. In addition, it is also necessary to pay attention to any motor or sensory dysfunction, self-care ability limitations, and nutritional status after discharge [55,56]. Carlson’s study [23] has shown that telemedical support from a multidisciplinary team can provide rehabilitation, nutrition suggestions, and psychological guidance as well as how to deal with the threat of pressure injury. When a discharged patient has health-related problems, they can get appropriate help in time. Effort should be devoted in clinical practice to promoting multidisciplinary team cooperation and comprehensively promoting the physical and mental recovery of patients with SCI.

The hospital stays of patients with SCI are shorter now than in the past [57,58]. That allows less time for patients to receive education, rehabilitation, and adjustment, making them more likely to benefit from subsequent telemedicine. In Vesmarovich’s study [50], the patients and their families were given guidance on dressing techniques before discharge, and video was used to give continued medical care after discharge. That improved the rate of pressure injury healing. Many primary care doctors lack the expertise and skills to deal with the complex needs of patients with SCI [59], but access to specialized rehabilitation institutions is costly and might be difficult to arrange. When patients have insufficient resources to cope with the disease, they are more likely to aggravate pressure injury [60]. Huang [46] reports using a combination of telemedicine and nontelemedicine techniques to help medical staff change pressure injury dressings during home visits. Families were trained by telephone. Home visits allow for face-to-face treatment of pressure injury and providing professional guidance. They can to some extent compensate for the reductions in education time caused by shorter hospital stays.

The results show that using telemedicine did not increase the economic burden of SCI. Most developed countries provide patients with SCI with any equipment they may need to cope with their injury. They receive training before discharge and then remote written or oral guidance without the need for professionals to enter the patient’s home. That helps to minimize the cost of an SCI [40]. A study by Xu [61] showed that telemedicine can save money without reducing efficacy. The studies included in this systematic review rarely discuss cost considerations. In most of them, the patients received any necessary equipment for free or at low cost. Future research should conduct a rigorous cost-benefit analysis to demonstrate not only the impact on patient health, but also the value of investing in telemedicine intervention.

The network meta-analysis showed that the best intervention for preventing pressure injury combined two or more forms of telemedicine. The most common combination was internet chat (usually WeChat) with telephone conversations. Patients and their carers cannot be assumed able to identify pressure injury early and take countermeasures soon enough of their own accord [40]. Professionals, though, can observe patients’ skin using pictures or video and provide timely medical advice, thereby reducing the incidence of pressure injury. At present, the diagnostic accuracy from using images compared with that achieved through face-to-face evaluation remains unclear. That needs further documentation in well-designed studies with large samples. Of course, even if patients and carers receive the knowledge they need, over time, that knowledge may well be gradually forgotten. In China, the typical caregiver is older than their patient. More than 30% of caregivers are over 60 years old [62]. Moreover, even some middle-aged persons cannot effectively use a smartphone and a networking platform. They prefer telephoning or outpatient follow-up [63]. Nevertheless, repeated instruction, whether by telephone or internet messaging, and regular push messages to remind caregivers, can effectively reduce the incidence of pressure injury [64]. Any telemedicine intervention should of course suit the individual patient’s needs, condition, home situation, and level of medical understanding.

### Limitations

This study was to some unknown extent restricted by being limited to reports in either Chinese or English. Beyond that, some experimental studies were not included because they were unfinished or the relevant data could not be extracted. That may induce a certain degree of publication bias. There were also reports that did not describe the intervention frequency in detail, and some in which the accuracy of individual outcomes was relatively low due to the small number of related studies. More high-quality RCTs with large samples are need for further demonstration.

### Conclusions

Current evidence shows that telemedicine is an economical and feasible form of intervention. It can reduce the incidence of pressure injury in community-dwelling patients with SCI. Combining telemedicine with other sorts of intervention is better than using telemedicine alone. Telemedicine can improve the rate of pressure injury healing and reduce the severity of the injury without increasing the medical economic burden on patients with SCI. These above conclusions need to be further verified by additional high-quality RCTs using large samples. Future studies could explore the research on telemedicine in languages other than Chinese and English.

## Appendix

Multimedia Appendix 1 Search details.

Multimedia Appendix 2 Characteristics of the studies included.

Multimedia Appendix 3 Risk of bias assessment summary for each Cochrane item.

Multimedia Appendix 4 Risk of bias assessment for the quasi-experimental studies.

Multimedia Appendix 5 The effectiveness of telemedicine on the incidence of pressure injury (quasi-experimental studies).

Multimedia Appendix 6 Node splitting results.

Multimedia Appendix 7 Relative treatment rankings.

Multimedia Appendix 8 A comparison-adjusted funnel plot of the studies.

## Abbreviations

PRISMA-NMA: Preferred Reporting Items for Systematic Reviews and Meta-Analyses extension for Network Meta-Analysis
PROSPERO: International Prospective Register of Systematic Reviews
PUSH: Pressure Ulcer Scale for Healing
RCT: randomized controlled trial
RR: relative risk
SCI: spinal cord injury
SUCRA: surface under the cumulative ranking
WMD: weighted mean difference
