# Supplementary material for: Telemedicine for Preventing and Treating Pressure Injury After Spinal Cord Injury: Systematic Review and Meta-analysis
Source: J Med Internet Res. 2022 Sep 7;24(9):e37618. doi: 10.2196/37618 (PMC9494222; doi:10.2196/37618)
Supplement: Multimedia Appendix 1 [file jmir_v24i9e37618_app1.docx]

**Multimedia Appendix 1.** Search details

**Scopus：**

( TITLE-ABS-KEY ( spinal AND cord AND injuries OR spinal AND cord AND diseases OR spinal AND cord AND dysfunction OR quadriplegia OR paraplegia OR tetraplegia ) AND TITLE-ABS-KEY ( pressure AND ulcer OR pressure AND injury OR pressure AND sore OR bedsore OR decubitus ) AND TITLE-ABS-KEY ( telecommunication OR telehealth OR telemedicine OR internet OR telerehabilitation OR telenursing OR ehealth OR mobile OR smart AND phone OR application OR social AND medial OR computer AND network OR software OR telephone OR videoconferencing OR wechat OR aftercare OR follow-up AND studies ) )

**Cochrane library:**

telemedicine OR internet OR software OR follow-up studies OR videoconferencing OR aftercare OR tele* OR internet* OR mhealth* OR APP OR application OR social medial OR WeChat in All Text AND spinal cord injur* OR spinal cord diseases OR spinal cord dysfunction OR quadriplegia OR paraplegia OR tetraplegia in All Text AND pressure injury OR pressure ulcer OR pressure sore OR bedsore OR decubitus in All Text

**Embase：**

(telemedicine:ti,ab,kw OR internet:ti,ab,kw OR software:ti,ab,kw OR 'follow-up studies':ti,ab,kw OR videoconferencing:ti,ab,kw OR aftercare:ti,ab,kw OR tele*:ti,ab,kw OR internet*:ti,ab,kw OR mhealth*:ti,ab,kw OR app:ti,ab,kw OR application:ti,ab,kw OR 'social medial':ti,ab,kw OR wechat:ti,ab,kw) AND ('spinal cord injur*':ti,ab,kw OR 'spinal cord diseases':ti,ab,kw OR 'spinal cord dysfunction':ti,ab,kw OR quadriplegia:ti,ab,kw OR paraplegia:ti,ab,kw OR tetraplegia:ti,ab,kw) AND ('pressure injury':ti,ab,kw OR 'pressure ulcer':ti,ab,kw OR 'pressure sore':ti,ab,kw OR bedsore:ti,ab,kw OR decubitus:ti,ab,kw)

**Pubmed：**

((((((("Telemedicine"[Mesh]) OR "Internet"[Mesh]) OR "Software"[Mesh]) OR "Follow-Up Studies"[Mesh]) OR "Aftercare"[Mesh]) OR (tele*[Title/Abstract] OR internet*[Title/Abstract] OR mhealth*[Title/Abstract] OR APP[Title/Abstract] OR application[Title/Abstract] OR social medial[Title/Abstract] OR WeChat[Title/Abstract])) AND ((((("Spinal Cord Injuries"[Mesh]) OR "Spinal Cord Diseases"[Mesh]) OR "Quadriplegia"[Mesh]) OR "Paraplegia"[Mesh]) OR (spinal cord dysfunction OR quadriplegia OR paraplegia OR tetraplegia))) AND (("Pressure Ulcer"[Mesh]) OR (pressure injury OR pressure sore OR bedsore OR decubitus))

**Web of Science：**

TOPIC: (“telemedicine” OR “internet” OR “software” OR “follow-up studies” OR “videoconferencing” OR “aftercare” OR “tele*” OR “internet*” OR “mhealth*” OR “APP” OR “application” OR “social medial” OR “WeChat”) AND TOPIC: (“spinal cord injur*” OR “spinal cord diseases” OR “spinal cord dysfunction” OR “quadriplegia” OR “paraplegia” OR “tetraplegia”) AND TOPIC: (“pressure injury” OR “pressure ulcer” OR “pressure sore” OR “bedsore” OR “decubitus”)

**Proquest:**

full:( telemedicine OR internet OR software OR follow-up studies OR videoconferencing OR aftercare OR tele* OR internet* OR mhealth* OR APP OR application OR social medial OR WeChat) and full:( spinal cord injur* OR spinal cord diseases OR spinal cord dysfunction OR quadriplegia OR paraplegia OR tetraplegia

) and full:( pressure injury OR pressure ulcer OR pressure sore OR bedsore OR decubitus)

**CNKI：**

TKA =('远程通信'+'远程医学'+'远程医疗'+'远程康复'+'远程护理'+'因特网'+'智能手机'+'软件'+'应用程序'+'社交媒体'+'电话'+'视频会议'+'微信'+'出院后医疗'+'随访研究') AND TKA=('脊髓损伤'+'脊髓疾病'+'四肢瘫'+'截瘫') AND TKA =('压疮'+'压力性溃疡''+'压力性损伤'+'褥疮')

**Wanfang：**

检索表达式（中英文扩展&主题词扩展）： 主题:(脊髓损伤 OR 脊髓疾病 OR 截瘫 OR 四肢瘫) and 主题:(压疮 OR 压力性溃疡 OR 压力性损伤 OR 褥疮) and 主题:(远程通信 OR 远程医学 OR 远程医疗 OR 远程康复 OR 远程护理 OR 因特网 OR 智能手机 OR 软件 OR 应用程序 OR社交媒体 OR 电话 OR 视频会议 OR 微信 OR 出院后医疗 OR 随访研究)

**Vip：**

文摘=脊髓损伤OR脊髓疾病OR截瘫OR四肢瘫 AND 文摘=压疮OR压力性溃疡OR压力性损伤OR 褥疮 AND 文摘=远程通信 OR 远程医学 OR 远程医疗 OR 远程康复 OR 远程护理 OR 因特网 OR 智能手机 OR 软件 OR 应用程序 OR社交媒体 OR 电话 OR 视频会议 OR 微信 OR 出院后医疗 OR 随访研究

**CBM：**

[( "脊髓损伤"[全部字段:智能] OR "脊髓疾病"[全部字段:智能] OR "截瘫"[全部字段:智能] OR "四肢瘫"[全部字段:智能]) AND( "压疮"[全部字段:智能] OR "压力性溃疡"[全部字段:智能] OR "压力性损伤"[全部字段:智能] OR "褥疮"[全部字段:智能]) AND( "远程通信"[常用字段:智能] OR "远程医学"[常用字段:智能] OR "远程医疗"[常用字段:智能] OR "远程康复"[常用字段:智能] OR "远程护理"[常用字段:智能] OR "因特网"[常用字段:智能] OR "智能手机"[常用字段:智能] OR "软件"[常用字段:智能] OR "应用程序 OR社交媒体"[常用字段:智能] OR "电话"[常用字段:智能] OR "视频会议"[常用字段:智能] OR "微信"[常用字段:智能] OR "出院后医疗"[常用字段:智能] OR "随访研究"[常用字段:智能])](javascript:toDoRelimitSearch();)
