# Supplementary material for: Telemedicine for Preventing and Treating Pressure Injury After Spinal Cord Injury: Systematic Review and Meta-analysis
Source: J Med Internet Res. 2022 Sep 7;24(9):e37618. doi: 10.2196/37618 (PMC9494222; doi:10.2196/37618)
Supplement: Multimedia Appendix 2 [file jmir_v24i9e37618_app2.pdf]

Multimedia Appendix 2. Characteristics of the studies included

Author, Year	Country	Sample size [a]	male/ female	Age[b] (years)	Tetraplegia/ paraplegia	level of injury [c]	Intervention modality	Control treatment	Study duration (months)	Outcome
Arora [7] 2017	India Bangladesh	60-57[d]/ 60-58[d]	52/8<br>54/6	35±11<br>36±12	17/37/6[e]<br>12/40/8[e]	47/11/2[e]<br>52/5/3[e]	SCTI<br>Telephone<br>Telephone once/week	BC	3	③④⑤⑥
Qiaoping Li [17] 2021	China	40/40	28/12<br>26/14	57.8, 23-72<br>59.1, 17-69	13/27<br>15/25	5/35<br>6/34	PTI<br>WeChat + QQ + telephone + outpatient follow up+ home visit<br>1 week, 4 months for home visit, 3 month for outpatient follow up	BC	12	①②
Jing Li 2021 [18]	China	39/39	40/38	47.12±10.90	10/68	25/53	SCTI<br>WeChat	BC	6	①
Hossain [19] 2020	Bangladesh	204-189[d]/ 206-189[d]	181/23<br>188/18	25.7- 45<br>24.5- 41	84/120<br>87/119	144/60<br>148/58	PTI<br>Telephone + home visit.<br>36 telephone calls and 3 home visits	BC	24	①③
Shengjie Zou [20] 2020	China	50/50	35/15<br>33/17	46.07±5.51<br>45.27±5.12	-	0/50<br>0/50	MCTI<br>WeChat + Telephone weekly<br>Consulting online 1 hour/day using WeChat	SCTI<br>Telephone once/week	3	①

Yuexi Chen [21] 2020	China	60/60	48/12<br>47/13	48.77±9.31<br>48.56±9.56	-	-	MCTI<br>Telephone + WeChat<br>1, 2 weeks, 1, 2, 6 months by telephone	BC	3	①
Kryger [22] 2019	USA	19/19	13/6<br>12/7	37.9±13.4<br>44.1±15.3	8/11<br>9/10	9/10<br>12/7	SCTI<br>iMHere app with functions such as drug and skin management providing, information	BC	9	②
Carlson [23] 2019	USA	82-73[df]<br>84-70[dg]<br>66[h]	69/13<br>72/12<br>60/6	41.7±12.9<br>42.5±12.2<br>44.0±14.0	21/58/3[e]<br>61/22/1[e]<br>46/12/8[e]	61/21<br>55/28/1[e]<br>49/17	MCTI<br>Home visit + telephone<br>1-6 months (once/week), 9 home visits, 15 telephone conversations; 7-12 months 2 home visits of 1.5h, 9 telephone consultations of 30 minutes<br>SCTI<br>Telephone once a quarter	BC	24	①
Xuanhui Dai [24] 2019	China	40/40	26/14<br>27/13	35.48±5.98<br>35.12±6.54	40/0<br>40/0	40/0<br>40/0	MCTI<br>WeChat + telephone<br>Consulting online 1 hour/day, twice weekly using WeChat	BC	6	①
Kun Zhao [25] 2018	China	52/52	27/25<br>28/24	35.59±4.41<br>35.55±4.42	4/46<br>7/45	-	PTI<br>Outpatient follow up telephone + home visits at	BC	6	①

							2, 4, 6, 8 weeks			
Jiefang Li [26] 2018	China	32/32	17/15<br>18/14	37.6±3.1<br>35.9±3.4	11/21<br>9/23	-	MCTI<br>Telephone +QQ+ WeChat<br>1, 2 weeks, then 1, 2, 6 months by telephone	BC	6	①
Hossain [27] 2017	Bangladesh	15-14[d]/<br>15-14[d]	13/2<br>13/2	29, 24-35<br>34, 23-36	5/10<br>6/9	12/3<br>12/3	SCTI<br>Telephone<br>twice a week for the first year, monthly the second year	BC	24	①③
Ying Yang [28] 2017	China	100/100	43/57<br>45/55	52.73±6.34<br>52.86±7.56	22/78<br>19/81	-	PTI<br>WeChat + telephone + texting + home visit	NTI<br>Outpatient follow up	6	①
Cuicui Li [29] 2017	China	30/30	24/6<br>25/5	41.16±11.35<br>43.31±12.48	30/0<br>30/0	12/18<br>10/20	MCTI<br>WeChat + telephone<br>Consulting online 1 hour/day plus twice weekly using WeChat	SCTI<br>Telephone<br>1 week, then 1, 3, 6 months	6	①
Li Ma [30] 2017	China	25/25	45/5	38, 22-62	6/40	-	MCTI<br>WeChat + telephone<br>2 weeks, then 1, 2, 3, 6, 12, 18 months by telephone	SCTI<br>2 weeks, then 1, 2, 3, 6, 12, 18 months by telephone	18	②
Xiaowei Luo [31] 2017	China	50/50	26/24<br>23/27	46.3±1.5<br>47.2±1.9	6/44<br>8/42	1/49<br>0/50	PTI<br>Telephone + outpatient follow up + home visits at<br>1, 4, 7, 10 months	BC	12	①

Wei Jing [32] 2017	China	32/32	19/13<br>18/14	57.5±5.1<br>56.9±5.4	9/23<br>10/22	32/0<br>32/0	PTI<br>Telephone+ home visit<br>2 weeks, then home visits at 1, 2, 3, 4, 5, 6 months	SCTI<br>3 times in the first month, then monthly by telephone	6	①
Yuhong Feng [33] 2017	China	37/ 35	54/18	39-78	0/37<br>0/35	-	MCTI<br>WeChat + Telephone<br>weekly in the first month, then twice monthly for 2 times months, monthly for 3 months), and semii-monthly for 6 months	BC	12	①
Xiuying Fan [34] 2017	China	40/40	18/22<br>19/21	30.2±10.8<br>31.7±10.1	0/40<br>0/40	3/37<br>2/38	PTI<br>Outpatient follow up at 1,4,7,10 month +WeChat	Outpatient follow up	12	①
Yanli Yang [35] 2017	China	32/32	19/13<br>18/14	36.5±8.95<br>37.1±8.65	0/32<br>0/32	-	PTI<br>Telephone + outpatient follow up at 1, 3, 6 weeks, then 4 months by telephone.	SCTI<br>Same without the outpatient follow up	6	①
Houlihan [36] 2013	New England	53/53	42/11<br>34/19	45.8±12.1<br>45.0±14.0	23/27/3[e]<br>28/25	-	SCTI<br>Telephone<br>CareCall (an automated interactive voice response system) weekly	BC	6	③

Zaihua Ru [37] 2013	China	12/12	9/3<br>10/2	42.3±12.0<br>45.3±11.4	3/9<br>4/8	10/2<br>9/3	PTI<br>Monthly home visit+ telephone at any time.	Home visit	12	①
Xiaoli Fan [38] 2011	China	18/18	12/6<br>11/7	37.2±7.2<br>38.5±7.6	2/16<br>2/16	-	PTI<br>Telephone + home visits at 2 weeks, and monthly for 6 months; telephone follow up at any time	BC	6	①
Dallolio [39] 2008	Italy, Belgium, England	62-53[d]/ 65-61[d]	53/6<br>12/5<br>42/9	37.34±13.64<br>37.88±15.41<br>43.90±15.75	24/35<br>3/14<br>20/28/3[e]	-	SCTI<br>Weekly video telemedicine for two months, then bi-monthly for four months; about 45 minutes at a time	BC	6	①
Phillips [40] 1999	USA	13[i]<br>12[j]<br>10[h]	9/4<br>9/3<br>8/2	29.6±6.4<br>33.4±13.8<br>38.1±15.2	-	-	SCTI<br>Weekly video and telephone for 10 to 12 weeks	BC	6–8	①
Li Ma [41] 2020	China	60	53/7	-	10/50	-	TI<br>WeChat.	Self-control	24	②
Qiaoling Chen [42] 2017	China	35/30	30/5<br>27/3	29.37±7.71<br>29.77±8.38	0/35<br>0/30	35/0<br>30/0	MCTI<br>QQ + WeChat + weekly themed activities; semi-weekly mailings; Face-to-face online 1 hour a week	BC	3	①

Jing Li [43] 2017	China	39/39	23/16<br>22/17	48.36±11.04<br>45.87±10.75	0/39<br>0/39	12/27<br>13/26	PTI<br>Weekly WeChat + monthly lecture + monthly patient association meeting + 1 home visit + weekly telephone	BC	3	①
Aihua Xu [44] 2016	China	90/90	54/36<br>48/42	43, 20-75	45/135	-	PTI<br>Telephone at 1 and 2 weeks, 1 and 2 months; home visit at 3 and 6 months	Outpatient follow up	6	①②
Ying Wang [45] 2015	China	28/28	38/18	37.05±9.43	20/36	56/0	PTI<br>Telephone + home visit 1, 2, 4 weeks, then monthly telephone for 5 months	SCTI<br>Telephone. 1, 2, 4 weeks, 2, 3, 4, 5, 6 months for telephone	6	①
Aihua Huang [46] 2014	China	36	29/7	37.74±2.31	0/36	15/21	TI<br>Semi-weekly telephone + semi-monthly home visits	Self-control	6	②
Xin He [47] 2014	China	29	12/17	68, 34-96	0/29	-	TI<br>Email + telephone	Self-control	2-11[k]	②
Tongxia Xia [48] 2012	China	40/40	30/10<br>30/10	18-65<br>19-64	0/40<br>0/40	-	SCTI<br>Weekly telephone for 8 weeks)	BC	3	①
Fang Lian [49] 2010	China	33/32	48/17	15-67	9/56	0/33<br>0/32	SCTI<br>Telephone at	BC	12	①②

Vesmarovich [50] 1999	USA	8	8/0	38-78	5/3	-	1 week, then at 1, 3, 6 and 12 months<br>TI<br>Weekly video	Self-control	1-18[k]	②

a:intervention group/control group; b: mean±SD or mean, min-max or min-max; c: complete/incomplete injury; d: Original sample size-sample size after loss of follow up; e: unknown classification; f: mixed complete telemedicine intervention group; g: single complete telemedicine intervention group; h: blank control; i: telephone group; j: video group; k: weeks; SCTI: single complete telemedicine intervention; BC: blank control; PTI: partial telemedicine intervention; MCTI: mixed complete telemedicine intervention; TI: telemedicine intervention; ① Incidence of pressure injury; ② Healing rate of pressure injury; ③ Pressure Ulcer Scale of Healing score; ④ The depth of pressure injury; ⑤ The size of pressure injury; ⑥ Economic data
