# Supplementary figures and images for: Telemedicine for Preventing and Treating Pressure Injury After Spinal Cord Injury: Systematic Review and Meta-analysis
Source: J Med Internet Res. 2022 Sep 7;24(9):e37618. doi: 10.2196/37618 (PMC9494222; doi:10.2196/37618)

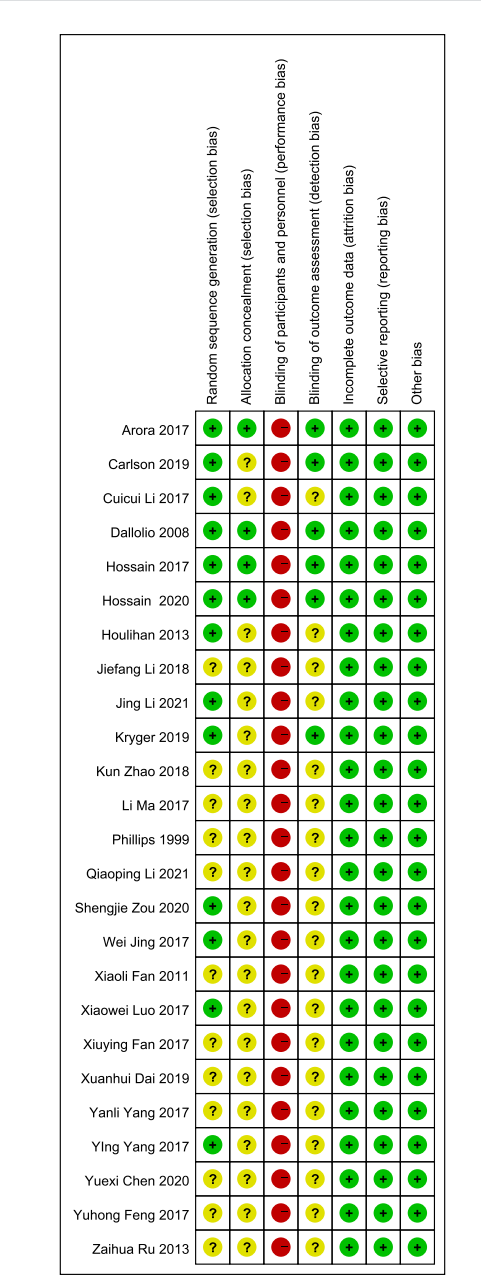


**Multimedia Appendix 3.** Risk of bias assessment summary for each Cochrane item

Supplement: Multimedia Appendix 3 [file jmir_v24i9e37618_app3.docx]
