# Supplementary material for: Telemedicine for Preventing and Treating Pressure Injury After Spinal Cord Injury: Systematic Review and Meta-analysis
Source: J Med Internet Res. 2022 Sep 7;24(9):e37618. doi: 10.2196/37618 (PMC9494222; doi:10.2196/37618)
Supplement: Multimedia Appendix 4 [file jmir_v24i9e37618_app4.docx]

**Multimedia Appendix 4. Risk of bias assessment for the quasi-experimental studies**

| Author, Year | Item 1 | Item 2 | Item 3 | Item 4 | Item 5 | Item 6 | Item 7 | Item 8 | Item 9 |
| --- | --- | --- | --- | --- | --- | --- | --- | --- | --- |
| Vesmarovich [50] 1999 | Y | Y | Na | Y | Y | Y | Y | Y | Y |
| Ma [41] 2020 | Y | Y | Na | Y | Y | Y | Y | Y | Y |
| Chen [42] 2017 | Y | Y | Y | Y | Y | Y | Y | Y | Y |
| Li [43] 2017 | Y | Y | Y | Y | Y | Y | Y | Y | Y |
| Xu [44] 2016 | Y | Y | Y | Y | Y | Y | U | Y | Y |
| Wang [45] 2015 | Y | Y | Y | Y | Y | Y | U | Y | Y |
| Huang [46] 2014 | Y | Y | Na | Y | Y | Y | Y | Y | Y |
| He [47] 2014 | Y | Y | Na | Y | Y | Y | Y | Y | Y |
| Xia [48] 2012 | Y | Y | Y | Y | Y | Y | U | Y | Y |
| Lian [49] 2010 | Y | Y | Y | Y | Y | Y | U | Y | Y |

Y=Yes, N=No, U=Unclear, Na=Not applicable
