# Supplementary material for: Telemedicine for Preventing and Treating Pressure Injury After Spinal Cord Injury: Systematic Review and Meta-analysis
Source: J Med Internet Res. 2022 Sep 7;24(9):e37618. doi: 10.2196/37618 (PMC9494222; doi:10.2196/37618)
Supplement: Multimedia Appendix 5 [file jmir_v24i9e37618_app5.docx]

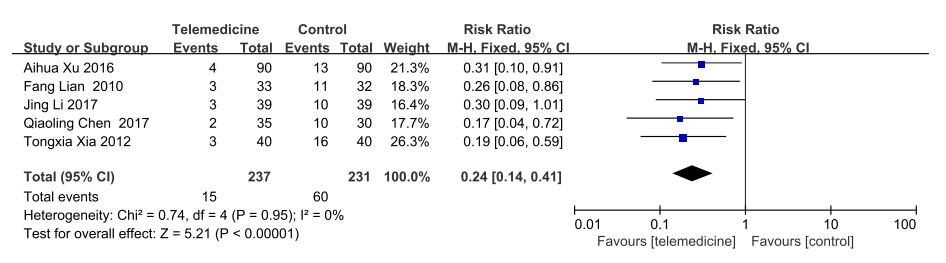
**Multimedia Appendix 5.** The effect of telemedicine on the incidence of pressure injury (quasi-experimental studies)
