# Supplementary material for: Telemedicine for Preventing and Treating Pressure Injury After Spinal Cord Injury: Systematic Review and Meta-analysis
Source: J Med Internet Res. 2022 Sep 7;24(9):e37618. doi: 10.2196/37618 (PMC9494222; doi:10.2196/37618)
Supplement: Multimedia Appendix 6 [file jmir_v24i9e37618_app6.docx]

**Multimedia Appendix 6.** Node-splitting results

| Side | Direct | | Indirect | | Difference | |  |
| --- | --- | --- | --- | --- | --- | --- | --- |
|  | Coeff. | Std. Err. | Coeff. | Std. Err. | Coeff. | Std. Err. | *p*>\|z\| |
| A C | -1.015888 | 0.557726 | 0.1928108 | 0.4419744 | -1.208699 | 0.7116177 | 0.089 |
| A D | -1.536316 | 0.5813942 | -1.529126 | 0.523956 | -0.0071892 | 0.7826552 | 0.993 |
| A E | -1.053188 | 0.2779898 | -2.322974 | 0.6725677 | 1.269786 | 0.7277539 | 0.081 |
| B E* | -0.9146971 | 0.4023264 | -2.286656 | 102.5846 | 1.371959 | 102.5862 | 0.989 |
| C D | -1.257636 | 0.332798 | -1.264677 | 0.7083855 | 0.0070413 | 0.782662 | 0.993 |
| C E | -1.609465 | 0.5184066 | -0.3392239 | 0.5108541 | -1.270241 | 0.7278202 | 0.081 |

A: Blank control; B: Non-telemedicine intervention; C: Single complete telemedicine intervention; D: Mixed complete telemedicine intervention; E: Partial telemedicine intervention;

*All of the evidence about these contrasts came from the trials which directly compared them;
