# Supplementary material for: Telemedicine for Preventing and Treating Pressure Injury After Spinal Cord Injury: Systematic Review and Meta-analysis
Source: J Med Internet Res. 2022 Sep 7;24(9):e37618. doi: 10.2196/37618 (PMC9494222; doi:10.2196/37618)
Supplement: Multimedia Appendix 7 [file jmir_v24i9e37618_app7.docx]

**Multimedia Appendix 7.** Relative treatment rankings

| Treatment | SUCRA | Pr. Best | Mean Rank |
| --- | --- | --- | --- |
| Blank control | 11.7 | 0.0 | 4.5 |
| Non-telemedicine intervention | 32.7 | 0.6 | 3.7 |
| Single complete telemedicine intervention | 31.7 | 0.0 | 3.7 |
| Mixed complete telemedicine intervention | 93.5 | 76.1 | 1.3 |
| Partial telemedicine intervention | 80.5 | 23.3 | 1.8 |
