# Supplementary material for: Telemedicine for Preventing and Treating Pressure Injury After Spinal Cord Injury: Systematic Review and Meta-analysis
Source: J Med Internet Res. 2022 Sep 7;24(9):e37618. doi: 10.2196/37618 (PMC9494222; doi:10.2196/37618)
Supplement: Multimedia Appendix 8 [file jmir_v24i9e37618_app8.docx]

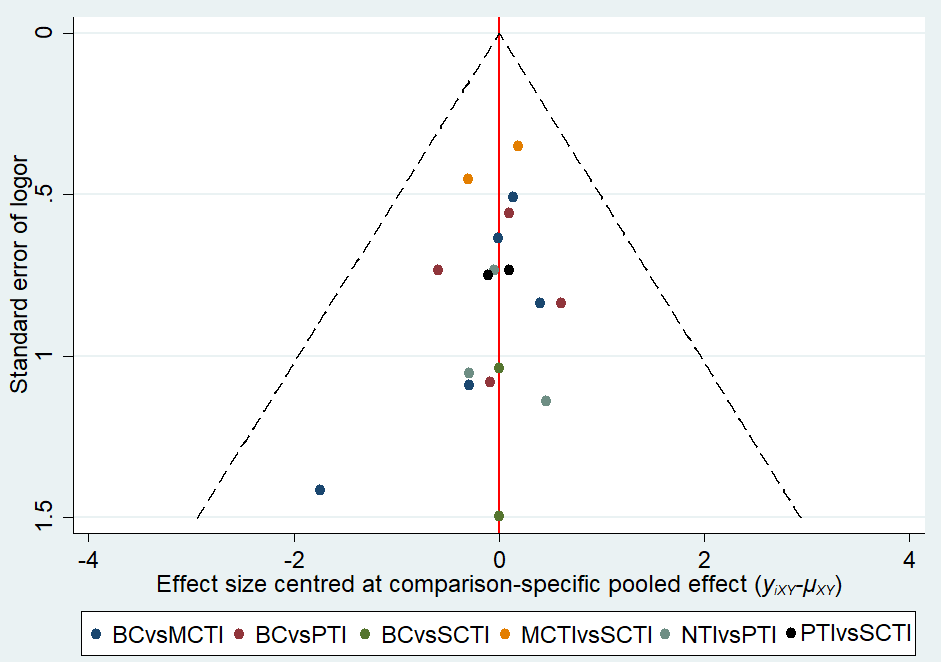


**Multimedia Appendix 8.** A comparison-adjusted funnel plot of the studies

BC: Blank control; MCTI: Mixed complete telemedicine intervention; PTI: Partial telemedicine intervention; SCTI: Single complete telemedicine intervention; NTI: Non-telemedicine intervention
